# Siblings in Flight: Unravelling Reproductive Barriers Between Two Butterfly Sister Species

**DOI:** 10.1002/ece3.74533

**Published:** 2026-11-08

**Authors:** Laura Torrado‐Blanco, Roger Vila, Vladimir A. Lukhtanov, Carmen Seoane‐Rodríguez, Camille Cornet, Kay Lucek, Marta Vila

**Affiliations:** ^1^ Departamento de Bioloxía Universidade da Coruña A Coruña Spain; ^2^ Institut de Biologia Evolutiva (CSIC‐Univ. Pompeu Fabra) Barcelona Spain; ^3^ Department of Karyosystematics, Zoological Institute of Russian Academy of Sciences Universitetskaya nab. 1 St. Petersburg Russia; ^4^ Biodiversity Genomics Laboratory, Institute of Biology University of Neuchâtel Neuchâtel Switzerland; ^5^ Grupo de Investigación en Bioloxía Evolutiva, Centro Interdisciplinar de Química e Bioloxía (CICA) Universidade da Coruña A Coruña Spain

**Keywords:** comparative genomics, endemism, female genitalia, introgression, karyotype, phylogeography, speciation, *Wolbachia*

## Abstract

Understanding how reproductive isolation is maintained between closely related taxa is a central question in evolutionary biology. Here, we investigate the mechanisms underlying divergence and isolation between two sister butterflies, *Erebia palarica* and *Erebia meolans*, which occur in sympatry in the Cantabrian Mountains (N Spain) and in allopatry elsewhere. Using an integrative approach, we combine genetic, genome synteny and cytogenetic analyses, *Wolbachia* screening, and geometric morphometrics to assess their evolutionary history and elucidate potential reproductive barriers between these species. The phylogeographic pattern suggests that both species diverged in allopatry, coming into secondary contact along the Cantabrian Mountains after the last glacial maximum. Both mitochondrial and nuclear markers recover reciprocal monophyly, indicating the absence of recent hybridisation. The identical chromosome numbers in both species (2*n* = 28), the absence of major chromosomal rearrangements or straightforward genitalia differentiation, and the presence of the same *Wolbachia* strain in all infected individuals suggest that neither chromosomal rearrangements nor *Wolbachia*‐induced incompatibilities contribute to their reproductive isolation in sympatry. In contrast, morphometric analyses show a significant divergence in wing size consistent with asymmetric character displacement: sympatric populations of *E. palarica* exhibit larger wings relative to both allopatric conspecifics and sympatric *E. meolans*, whereas *E. meolans* shows little size variation across northern Spain. Our findings point to wing size divergence in *E. palarica* as a response to sympatry with *E. meolans*. Overall, this study provides novel empirical evidence for morphometric character displacement in the family Nymphalidae, highlights the importance of multidisciplinary approaches for understanding species differentiation, and sets a framework for future genomic studies of speciation and reproductive isolation between closely related taxa.

## Graphical abstract

Using an integrative approach, we combine genetic, genome synteny and cytogenetic analyses, *Wolbachia* screening and morphology to assess the evolutionary history of two butterfly sister species (*Erebia palarica* and *Erebia meolans*) and elucidate their potential reproductive barriers.

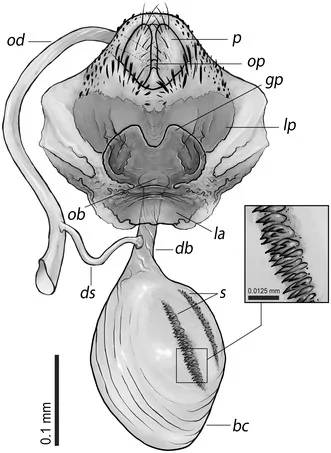

## 1 Introduction

Speciation in animals has traditionally been viewed as a linear process of lineage divergence. However, the splitting of genetic lineages often involves periods of secondary contact, during which hybridisation may occur (Augustijnen et al. 2022; Camizuli et al. 2026; Coyne and Orr 2004; Ebdon et al. 2025). In such cases, the persistence of species boundaries depends on the evolution and maintenance of effective reproductive barriers. Hybridisation is not uncommon among insects; for example Mallet (2005) estimated that at least 12% of European butterfly species show evidence of hybridisation with at least one other species. In this context, hybrid zones play a central role in speciation research, as they provide direct insight into the strength and mechanisms of reproductive isolation and reveal whether divergence is maintained, eroded, or reinforced following secondary contact (Barton and Hewitt 1989).

When hybridisation is maladaptive (e.g., hybrids are inviable or sterile, or exhibit reduced fitness), selection may favour mechanisms that reduce interspecific mating, giving rise to prezygotic and/or postzygotic barriers or strengthening existing ones (Abbott et al. 2013; Coyne and Orr 2004; Oliver 1979; Reifová et al. 2023). One possible outcome is character displacement (Brown and Wilson 1956), the biogeographic pattern of increased phenotypic differentiation between sympatric species or populations in response to ecological or reproductive interference. The term has been used inconsistently in the literature (Cooley 2007; Futuyma 1998; Pfennig and Pfennig 2009), often causing confusion. Here, we follow Howard (1993) and use character displacement and reinforcement to refer to the biogeographic pattern and the evolutionary process underlying it, respectively. In addition to morphological and behavioural mechanisms, the bacterial endosymbiont *Wolbachia* Hertig 1936 may also contribute to reproductive isolation in butterflies and other arthropods, as the infection by different *Wolbachia* strains can induce cytoplasmic incompatibilities that limit or prevent genetic exchange between host lineages (reviewed in Sasaki and Ishikawa 1999; Nice et al. 2009; Kaur et al. 2021; Wendt et al. 2022). Butterflies (Lepidoptera: Papilionoidea) constitute particularly suitable models for studying speciation and reproductive isolation, owing to their ecological diversity, repeated responses to geographic isolation during Pleistocene glacial cycles (Ebdon et al. 2021), plastic genomic architecture (de Vos et al. 2020), and frequent formation of hybrid zones (Gauthier et al. 2020; Hinojosa et al. 2024; Mallet 2005; Martins et al. 2023; Pateman et al. 2022). Speciose groups such as the genus *Erebia* Dalman, 1816 (Nymphalidae: Satyrinae) are particularly informative in this respect. This genus comprises over a hundred species (Peña et al. 2015; Tennent 2008), most of which are cold‐adapted, and exhibit substantial intraspecific variation in wing size, wing pattern and genital morphology (Chapman 1898; Jospin et al. 2023; Panigaj et al. 2015; Peña et al. 2015; Vila and Björklund 2004; Warren 1936). Variation within *Erebia* also extends to chromosome number, which ranges from *n* = 7 to *n* = 52 and represents a key driver for the diversification in this genus (Augustijnen et al. 2024).

Despite frequent sympatry among closely related and phenotypically similar species, as well as previous reports of hybridisation (reviewed in Jospin et al. 2023), many sister species of *Erebia* remain reproductively isolated. Here, we studied the case of *Erebia meolans* (Prunner, 1798) and *Erebia palarica* Chapman, 1905, two remarkably similar sister species that exhibit very low mitochondrial divergence: 1.6% in the most variable region of the *COI* gene (Vila and Björklund 2004), 0.61% in the *COI* barcode fragment (Dincă et al. 2021). *E. meolans* (the Piedmont ringlet) is broadly distributed across central and south‐western Europe (García‐Barros et al. 2013), whereas *E. palarica* (Chapman's ringlet) is a narrow endemic limited to the Cantabrian Mountains and adjacent mountain ranges of north‐western Spain (Figure 1). *E. palarica* coexists with *E. meolans* at the western end of the Cantabrian Mountains, and their flight periods overlap in summer (García‐Barros et al. 2013). Phylogenetic studies consistently recover the two species as sister taxa (Augustijnen et al. 2024; Peña et al. 2015; Wiemers et al. 2020) and highlight their strong phenotypic similarity, which makes them difficult to distinguish even on male genitalia morphology (García‐Barros et al. 2013).

**FIGURE 1:**
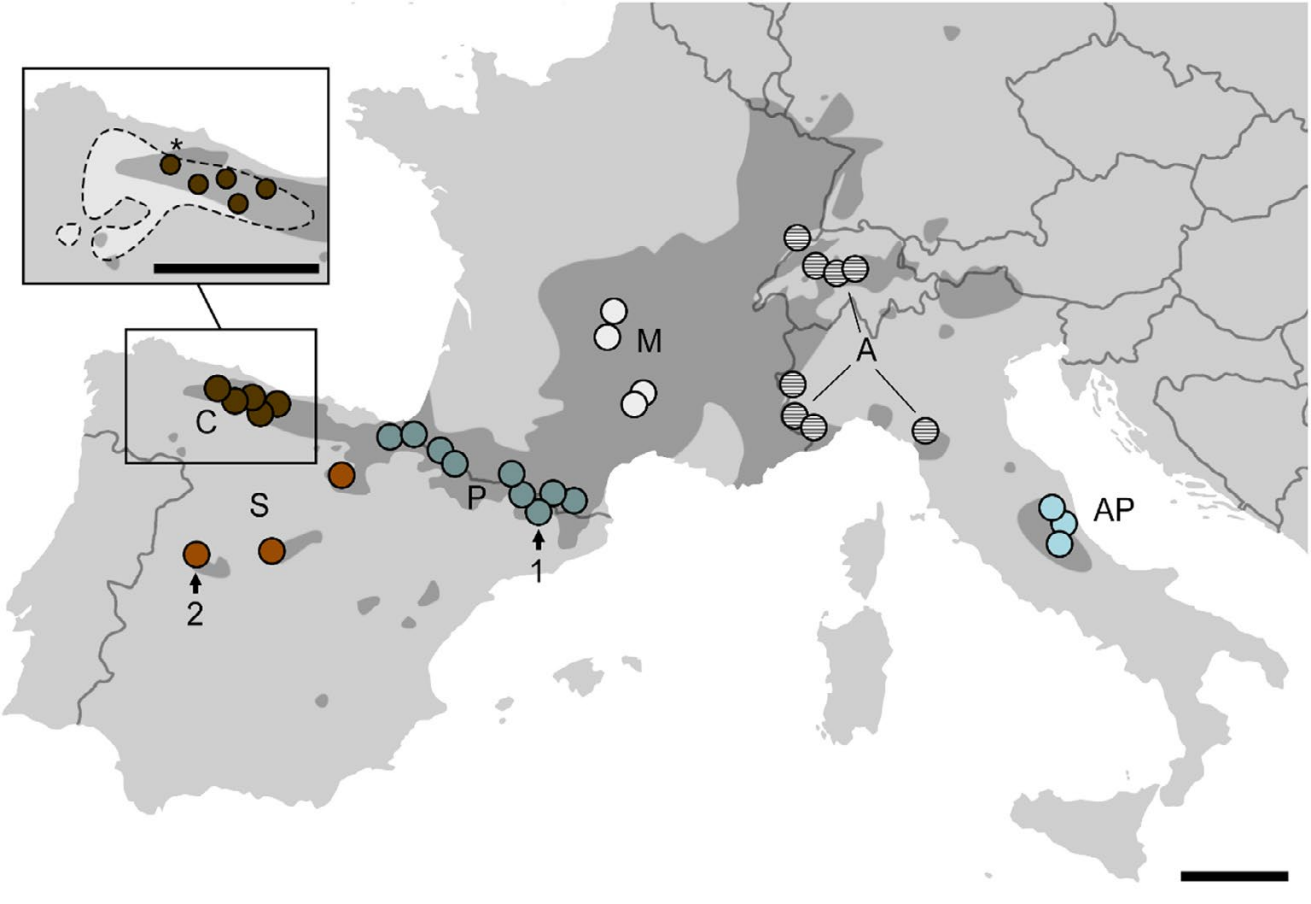
Overview of the localities (points) where *Erebia meolans* samples were collected: Cantabrian Mountains (C), Central System (S), Pyrenees (P), Massif Central (M), Alps (A) and Apennines (AP). Shaded areas indicate the approximate current distribution of *E. meolans* according to Sonderegger (2005, Switzerland), García‐Barros et al. (2013, Spain), Reinhardt et al. (2020, Germany), Oreina Association (2023, France) and Palmi (2023, Italy). The arrows highlight Coll de Jou (↑_1_) and Sierra de Béjar (↑_2_). The inset shows a detailed view of the contact zone between *E. meolans* (shaded) and *E. palarica* (dotted line); the asterisk (*) highlights the locality of Somiedo. Black bars represent 200 km; colours and geographic regions correspond to those shown in the haplotype network. Note that some localities were pooled to avoid visual overcrowding.

In this study, we combine genetic, genome synteny, cytogenetic and morphological analyses to disentangle the evolutionary history and reproductive isolation of *E. palarica* and *E. meolans*. We assess evidence of gene flow between the two species and examine potential reproductive barriers, including *Wolbachia* infection, chromosomal rearrangements and differences in female genitalia. In addition, we compare their phylogeographic pattern to determine whether their current sympatry in northern Spain results from secondary contact and evaluate whether a scenario consistent with reproductive character displacement contributes to their genetic isolation.

## 2 Methods

### 2.1 Molecular Analyses

#### 2.1.1 Sample Collection

We collected individuals from across their complete distribution ranges, that is Iberia and central‐western Europe. In total, we gathered 191 individuals of *Erebia meolans*, collected between 2000 and 2022: 168 from Spain, 7 from France, 10 from Italy and 6 from Switzerland (Figures 1 and 2, Supporting Information 1). Sampling followed the same procedures described in Torrado‐Blanco et al. (2024). For *E. palarica*, we included 305 specimens from 23 sites in NW Spain (Torrado‐Blanco et al. 2024). Due to their heterogeneous nature (i.e., differences in the type, quality and quantity of the tissue available for each individual), not all 496 samples were used in every analysis. See subsections and Supporting Information 1 for further details.

**FIGURE 2:**
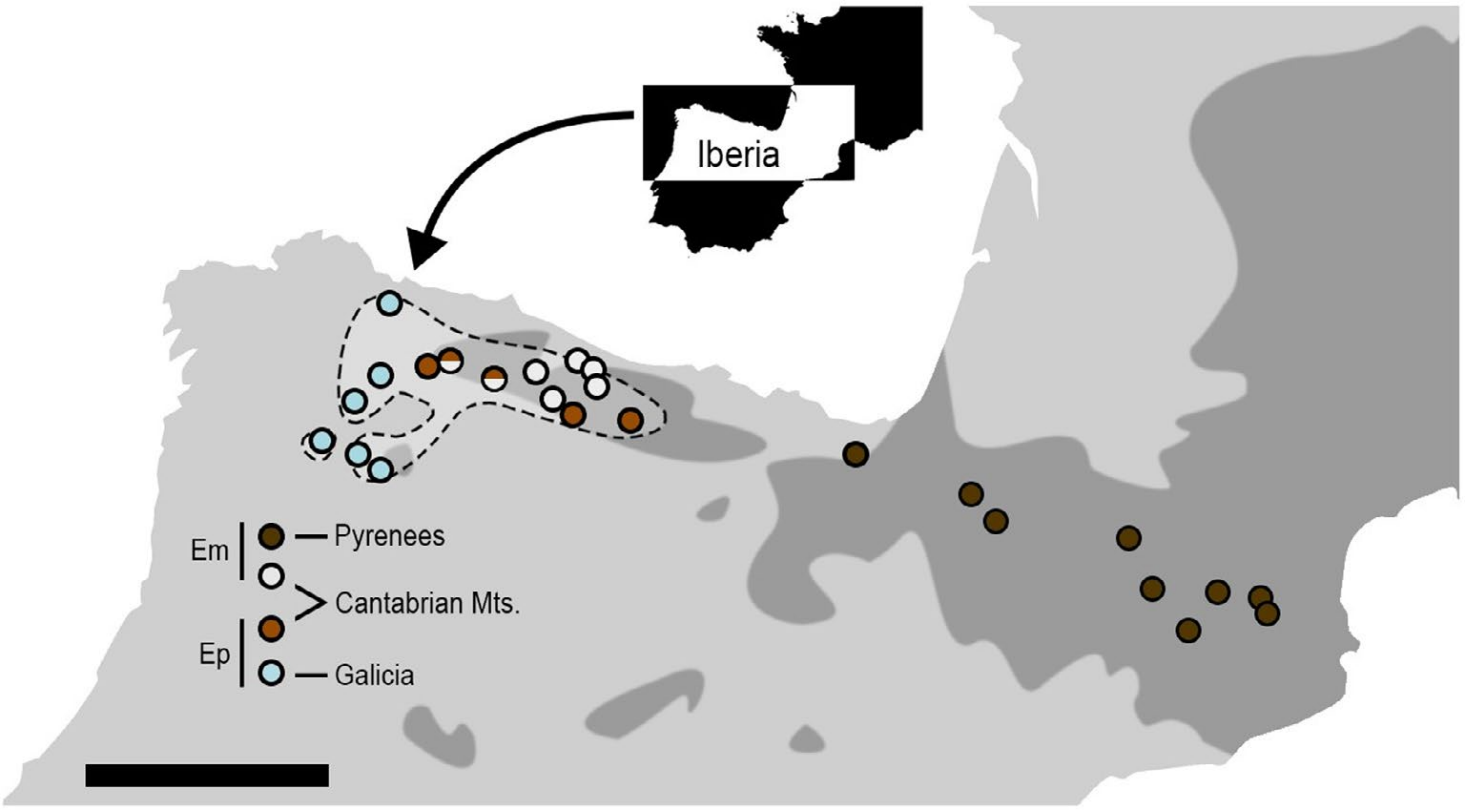
Detailed map showing the localities where *Erebia meolans* (Em) and *Erebia palarica* (Ep) were sampled in the Iberian Peninsula. Colours indicate the four species x region groups used in the morphometric analyses, which correspond to those shown in Figure 7. The distribution areas of both species are indicated by the shaded areas (*E. meolans*) and dashed line (*E. palarica*). The black bar represents 200 km.

Outgroup species for phylogenetic analyses were selected according to the phylogeny produced by Augustijnen et al. (2024) and encompass one specimen of *E. triaria* (Prunner, 1798), one of *E. euryale* (Esper, 1805), and one of *E. fletcheri* Elwes, 1899. *E. triaria* and *E. fletcheri* belong to the same clade as *E. palarica* and *E. meolans*, that is the *medusa* clade. Complementarily, *E. euryale* belongs to the more distant *ligea* clade.

#### 2.1.2 DNA Extraction

The study was built upon tissue samples of varying nature, collected by several researchers using different protocols and at different times, resulting in a heterogeneous dataset. Genomic DNA of Spanish *E. meolans* was extracted from thorax tissue using various kits—High Pure PCR Template preparation kit (Roche), Genelute Mammalian Genomic DNA Miniprep kit (Sigma‐Aldrich) and DNeasy‐Tissue‐Kit (Qiagen)—following the instructions provided by each manufacturer. DNA extractions of Central European (i.e., France, Italy and Switzerland) *E. meolans* and one *E. triaria* were obtained from thorax tissue using the High Pure PCR Template preparation kit (Roche). Extraction of the *E. palarica* samples is described in Torrado‐Blanco et al. (2024).

All the aforementioned DNA extractions were used for the phylogeographic study. Endosymbionts like *Wolbachia* tend to be more prevalent in gonads and Malpighian tubules in some insect groups (Faria and Sucena 2013). However, the leg and thorax tissue used for DNA extractions for the phylogenetic analyses did not always originate from complete individuals, meaning that corresponding abdominal tissue was not available for all specimens included in the phylogenetic dataset. Therefore, we performed 40 additional DNA extractions using abdomens of both species from different localities: (i) five individuals from Galician locations where *E. palarica* occurs in allopatry, (ii) five individuals from the Pyrenees where *E. meolans* occurs in allopatry, and (iii) 15 *E. palarica* and 15 *E. meolans* from three Cantabrian sites where both species co‐occur. We used the MicroSpin Genomic DNA kit (REAL) and the EZNA tissue DNA Purification kit (Omega Bio‐tek), following the manufacturer's protocols.

#### 2.1.3 PCR and Sequencing

To investigate the phylogenetic relationship between *E. meolans* and *E. palarica*, we followed Hinojosa et al. (2019) and analysed two types of molecular markers: (i) part of the mitochondrial Cytochrome Oxidase I gene (*COI*) and (ii) part of the nuclear gene *wingless*. Here, we targeted the second half of the *COI* gene (786 bp), as it provides greater resolution than the barcode fragment at intraspecific level (Lunt et al. 1996).

We first obtained the mitochondrial *COI* fragment for 305 individuals of *E. palarica* (retrieved from Torrado‐Blanco et al. 2024) and 168 individuals of Spanish *E. meolans*. Then, we selected specimens representing unique *COI* Spanish haplotypes and amplified the nuclear marker *wingless* only in those representatives. Lastly, we amplified both markers (*COI*, *wingless*) in 20 *E. meolans* from Central Europe.

*COI* amplifications were performed in 20 μL volumes containing 1× Buffer (5 PRIME), MgCl_2_ 2.5 mM, 1U DNA Taq‐polymerase (5 PRIME), 0.2 mM of each dNTP, 0.2 μM of each primer LCO1490 (Folmer et al. 1994) + Lep‐LEUr (Vila and Björklund 2004) and 50–400 ng of DNA. Reactions were conducted using a first step of 2 min at 95°C, followed by 35 cycles of 1 min at 94°C, 1 min 30 s at 57°C and 1 min 30 s at 68°C, and a final elongation phase of 7 min at 68°C.

*Wingless* amplifications were performed in 20 μL volumes containing 1× Buffer (5 PRIME), MgCl_2_ 2.5 mM, 1U DNA Taq‐polymerase (5 PRIME), 0.2 mM of each dNTP, and 0.25 μM of each primer LepWG1 + LepWG2 (Brower and DeSalle 1998) and 100–400 ng of DNA. We tried a PCR program with an initial step of 2 min at 95°C, followed by 40 cycles of 1 min at 95°C, 1 min at 51°C and 1 min 30 s at 68°C, and an elongation phase of 6 min at 68°C to amplify the target DNA of *E. meolans* from Central Europe. These settings were not optimal for Spanish samples of neither *E. meolans* nor *E. palarica*, as revealed by the presence of several unspecific bands in electrophoresis gels. Thus, we adjusted the parameters of the PCR program to an initial step of 2 min at 95°C, followed by 35 cycles of 1 min at 95°C, 45 s at 55°C and 45 s at 68°C, and an elongation phase of 6 min at 68°C, and successfully amplified the *wingless* fragment in those samples. PCR products were assessed on 2% agarose gels and visualised under UV light after RedSafe (iNtRON Biotechnology, South Korea) staining.

Amplified fragments were purified with ExoSAP‐IT (ThermoFisher Scientific) and bidirectionally sequenced on a 3500xl Genetic Analyser (Applied Biosystems, ABI) at Servizos de Apoio á Investigación (University of A Coruña). *COI* amplicons were sequenced with SeqIntCOIF (Vila and Björklund 2004) and Lep‐LEUr as forward and reverse primers, respectively. Conversely, PCR products of the *wingless* fragment were sequenced with the same primer pair used for amplification (LepWG1 + LepWG2). Raw data was manually inspected in codoncode aligner v.10.0.2 (CodonCode Corporation 2021). We used the option phase implemented dnasp v.6.12.03 (Rozas et al. 2017) to determine the most probable haplotype in nuclear sequences that carried two polymorphic sites (i.e., double heterozygotes). Curated sequences, aligned and collapsed into unique haplotypes, are deposited on GenBank (Clark et al. 2016; Table S1).

Finally, we amplified two coding fragments of the *Wolbachia* genome: ca. 590 bp of the *Wolbachia* surface protein (*wsp*) and ca. 470 bp of the aspartyl/glutamyl‐tRNA amidotransferase subunit B (*gatB*). Amplification reactions for *wsp* were performed in 20 μL volumes containing 1× Supreme NZYTaq II 2× Colourless Master Mix (NZYTech, Portugal), 0.20 μM of each primer wsp81F + wsp691R (Zhou et al. 1998) and 20 ng of DNA. The thermal cycling profile was initiated with a denaturing step (2 min at 95°C), followed by 38 cycles of 30 s at 95°C, 30 s at 55°C and 2 min at 72°C, and finished with an elongation phase of 2 min at 72°C. Reactions for *gatB* were prepared in 20 μL volumes containing 1× Supreme NZYTaq II 2× Colourless Master Mix (NZYTech, Portugal), 1 μM of each primer gatB_F1 + gatB_R1 (Baldo et al. 2006) and 20 ng of DNA. The thermal cycling profile was initiated with a denaturing step (2 min at 95°C), followed by 36 cycles of 30 s at 95°C, 30 s at 54°C and 1.5 min at 72°C, and finished with an elongation phase of 10 min at 72°C. All PCRs included the two positive controls for *Wolbachia* infection already used by Marí‐Mena et al. (2016). Post‐PCR steps were the same as described for *wingless*, that is PCR primers were also used for bidirectional sequencing.

#### 2.1.4 Diversity and Phylogeny

Standard indexes of genetic variation—number of haplotypes (*h*), number of segregating sites (*S*), number of parsimony informative sites (*S*′), haplotype diversity (*H*_d_), nucleotide diversity per gene (*π*), average number of nucleotide differences (*k*) and the *F*_ST_ nucleotide‐based statistic (Hudson et al. 1992; *F*_ST_ hereafter) were estimated with dnasp v.6.12.03 (Rozas et al. 2017).

We investigated the mitochondrial (*COI*) phylogeographic pattern of *E. meolans* and *E. palarica* with a 95% statistical parsimony haplotype network computed on tcs v.1.21 (Clement et al. 2000). The output was further optimised with popart (Leigh and Bryant 2015). Then, we reconstructed the phylogeny of the nuclear (*wingless*) and concatenated datasets of unique sequences using both maximum likelihood (ML) and Bayesian inference (BI). ML trees for *wingless* and the concatenated *COI* + *wingless* were computed in phyml v.3.0 (Guindon et al. 2010), selecting the HKY + I and TN93 + *R* models, respectively, as indicated by the Smart Model Selection (SMS) algorithm implemented in phyml (Lefort et al. 2017). We used 1000 bootstrap replicates to estimate branch support, setting all other parameters to their default values. The Bayesian phylogeny was calculated with mrbayes v.3.2.7a (Ronquist et al. 2012). We ran jmodeltest v.2.1.10 (Darriba et al. 2012) to select the best substitution model for the nuclear (HKY + I) and mitochondrial (HKY + I + G) datasets according to the Bayesian information criterion. This information was indicated for each partition in the analysis of the concatenated dataset. Given the short length of the alignments, we did not partition by codon position, as this would produce partitions too small for reliable parameter estimation. Two independent analyses of four Markov Chain Monte Carlo (MCMC) simulations were run in parallel for 1 (*wingless*) and 10 (*COI* + *wingless*) million generations. Convergence and stationarity were reached with default values for sampling and burn‐in. Convergence was indicated by the average standard deviation of splits (< 0.01), whereas stationarity was confirmed by a white noise plot of loglikelihood scores and PSRF values of 1.000 for all parameters. Results were presented as the halfcompat posterior probability consensus tree. All trees were visualised using presto (ATGC Montpellier Bioinformatics Platform 2024).

### 2.2 Cytological Analysis

We arranged chromosome preparations for 25 *E. palarica* and 11 *E. meolans* adult males (Table S2), processing them following Vershinina and Lukhtanov (2010). In brief, gonads were removed from the abdomen and placed into freshly prepared fixative (3:1; 96% ethanol and glacial acetic acid) directly after capturing the butterfly in the field. Testes were stored in the fixative for 6–12 months at +4°C. Then, the testes were stained in 2% acetic orcein for 10–30 days at +18°C–20°C. Chromosomes were analysed at the stage of mitosis and male atypical meiosis (Lorković 1990), which represents a type of asynaptic meiosis and displays unpaired chromosome homologues (Lukhtanov et al. 2020).

At the stage of atypical meiosis, chromosomes do not form a metaphase plate and are distributed throughout the volume of the atypical spermatocyte. Thus, the chromosomes or groups of chromosomes were photographed separately using manual scanning. The resulting layers with images of individual chromosomes (groups of chromosomes) were manually stacked in Photoshop.

### 2.3 Synteny

We further tested for potential large‐scale chromosomal rearrangements (e.g., inversions, fusions, fissions) between the reference genomes of *E. meolans* (Vila et al. 2025) and *E. palarica* (Vila et al. 2026). We first masked repetitive regions in the genomes using repeatmasker v4.1.5 (Smit et al. 2013) and the curated Dfam v3.7 Arthropoda repeat library (Storer et al. 2021), before gene prediction and annotation with braker2 v3.0.7 (Brůna et al. 2021) and the Arthropoda OrthoDB v11 protein set. We then ran orthofinder v2.5.5 (Emms and Kelly 2019) to identify orthologous genes between the two species, filtering the gene annotation to retain only the longest transcript per gene. To obtain a synteny plot and blocks of syntenic genes, we used genespace v1.3.1 (Lovell et al. 2022), which uses both orthologous information from orthofinder and reciprocal blast hits from mcscanx (Wang et al. 2012).

### 2.4 Morphometric Analyses

#### 2.4.1 Dataset and Photography

Wings from 59 *E. meolans* (14 ♀, 45 ♂) and 54 *E. palarica* (15 ♀, 39 ♂)—collected at 16 and 11 localities, respectively—were mounted on 45 × 60 mm glass slides. This dataset focused on the distribution of *E. meolans* along North Iberia, accounting for those areas where the species occurs in sympatry with *E. palarica* (i.e., the Cantabrian Mountains) as well as for allopatric sites (i.e., the Pyrenees). In the case of the endemic *E. palarica*, samples were representative of the complete distribution range (Figure 2).

The dorsal side of the left anterior wing of each butterfly was photographed following Torrado‐Blanco et al. (2024). Mirrored right wings were used in case the left wing was damaged (17 *E. meolans* and 28 *E. palarica*). Images were digitised with tpsdig v.2.32 (Rohlf 2021) and the resulting files were prepared for further geometric morphometric analyses (Zelditch et al. 2012) with tpsutil v.1.82 (Rohlf 2022). We defined a single, continuous, non‐overlapping curve tracing the complete outline of each wing from the base in a counter‐clockwise direction. Curves were re‐sampled for 70 homologous points (semilandmarks).

In addition, we dissected the genitalia of 16 individuals from sympatric and allopatric localities in northern Spain (*E. meolans*: 4 ♀, 4 ♂; *E. palarica*: 4 ♀, 4 ♂) and compared the anatomy of male and female dissections between the species pair (females: ductus and bursa length, males: valve, uncus and aedeagus length). To improve the visualisation of female structures, we submerged the tissue for 5–10 s in a few drops of a 1% chlorazol black solution. Samples were mounted on a microscope calibration slide and subsequently photographed under the stereomicroscope. Precise measures of male and female structures were extracted using tpsdig; see Supporting Information 1, tab Genitalia for further details.

#### 2.4.2 Data Analyses

We used the package *geomorph* (Adams et al. 2022) in R v.4.1.0 (R Core Team 2021) to analyse the morphometric data following Torrado‐Blanco et al. (2024). In short, we performed a Generalised Procrustes Analysis (GPA) on the semilandmarks to (i) compare wing shape between *E. meolans* and *E. palarica* and (ii) extract centroid sizes to investigate size variation.

A preliminary principal component analysis (PCA) using the Procrustes coordinates revealed the most informative axes regarding morphological variation. We selected seven principal components (PCs), accounting for 95.4% of cumulative variance in our dataset. We used multivariate analyses of variance (MANOVA) on the retained PCs to evaluate the effect of the variables of interest (i.e., species, sex and geographic region) over wing shape in *E. meolans* and *E. palarica*; we also performed a multivariate analysis of covariance (MANCOVA) considering species as covariate. Lastly, we used a canonical variate analysis (CVA) to visualise group differences. Three geographic regions were defined: the Cantabrian Mountains, the Pyrenees and Galicia (which includes the Galician Massif and Montes de León).

To investigate size variation, we compared centroid sizes in relation to (i) species, (ii) sex and (iii) geographic region (Supporting Information 1). Differences in centroid size were assessed via *t*‐tests for sex and species and a one‐way ANOVA with a Tukey HSD post hoc test for geographic region. Samples from Coll de Jou (Figure 1, ↑_1_) seemed to be a distinct unit based on exploratory analyses, but we considered them within the Pyrenees region based on geographic proximity and because they did not obscure the detected pattern.

## 3 Results

### 3.1 Molecular Analyses

#### 3.1.1 Genetic Diversity

We obtained high quality *COI* sequences (786 bp) for 188 *E. meolans* samples—168 from Spain and 20 from Central Europe. In addition, we produced *wingless* sequences (400 bp) for 43 *E. meolans*—24 from Spain and 19 from Central Europe—and 22 *E. palarica*.

The mitochondrial *COI* gene revealed 36 and 24 haplotypes for *E. meolans* and *E. palarica*, respectively. Complementarily, the gene *wingless* yielded five (*E. meolans*) and four (*E. palarica*) nuclear haplotypes (Table 1). A congruent diversity pattern was found for intraspecific haplotype diversity (*H*_d_): *E. meolans* (*COI*: 0.838; *wingless*: 0.474) and *E. palarica* (*COI*: 0.701; *wingless*: 0.544). The number of polymorphic sites (*S*) is almost equal to the number of haplotypes for each gene and taxon, indicating that each haplotype differs from others in just one or a few sequence positions. The average number of nucleotide differences (*k*) is congruent, showing values of 2.611 (*COI*) and 0.506 (*wingless*) for *E. meolans*, and 1.168 (*COI*) and 0.594 (*wingless*) for *E. palarica*. Nucleotide diversity was small for both genes and species (*π* = 0.001–0.003), where *F*_ST_ between *E. meolans* and *E. palarica* is remarkably high for both *COI* (0.847) and *wingless* (0.922).

**TABLE 1:** Genetic diversity of *Erebia meolans* (Em) and *Erebia palarica* (Ep) as given by the most variable region of the cytochrome oxidase I gene (*COI*, 786 bp) and the nuclear gene *wingless* (*wg*, 400 bp).

Gene	Species	*N*	*h*	*S*	*S*′	*H*_d_ (±SD)		*π* (±SD)		*k*	*f*_st_
*COI*	Em	188	36	38	35	0.838	0.001	0.003	0.0002	2.611	0.847
	Ep	305	24	24	13	0.701	0.018	0.001	0.0001	1.168	
*wg*	Em	43	5	4	1	0.474	0.042	0.001	0.0001	0.506	0.922
	Ep	22	4	3	2	0.544	0.052	0.001	0.0002	0.594	

*Note:* Abbreviations refer to the number of sequences (*N*), number of haplotypes (*h*), number of segregating sites (*S*), parsimony informative sites (*S*′), haplotype diversity (*H*_d_), nucleotide diversity (*π*), average number of nucleotide differences (*k*) and fixation index (*F*_ST_). Standard deviations are indicated as SD.

#### 3.1.2 Phylogeny

The topology produced both by ML and BI for the nuclear gene *wingless* shows a highly supported reciprocal monophyly for *E. palarica* and *E. meolans* (Figure 3A). The concatenated tree of the nuclear and mitochondrial sequences (Figure 3B, Figures S1 and S2) further supports this result. Despite the large polytomy recovered for each species, a few supported clades arise: *E. meolans* samples from Sierra de Béjar (central Iberia; Figure 1, ↑_2_) and from the Central Apennines (Italy; Figure 1 AP). In the case of *E. palarica*, a large polytomy includes most individuals except for some sequences from Picos de Europa and Serra de Ancares (East and West Cantabrian Mountains, respectively; Figure 1C).

**FIGURE 3:**
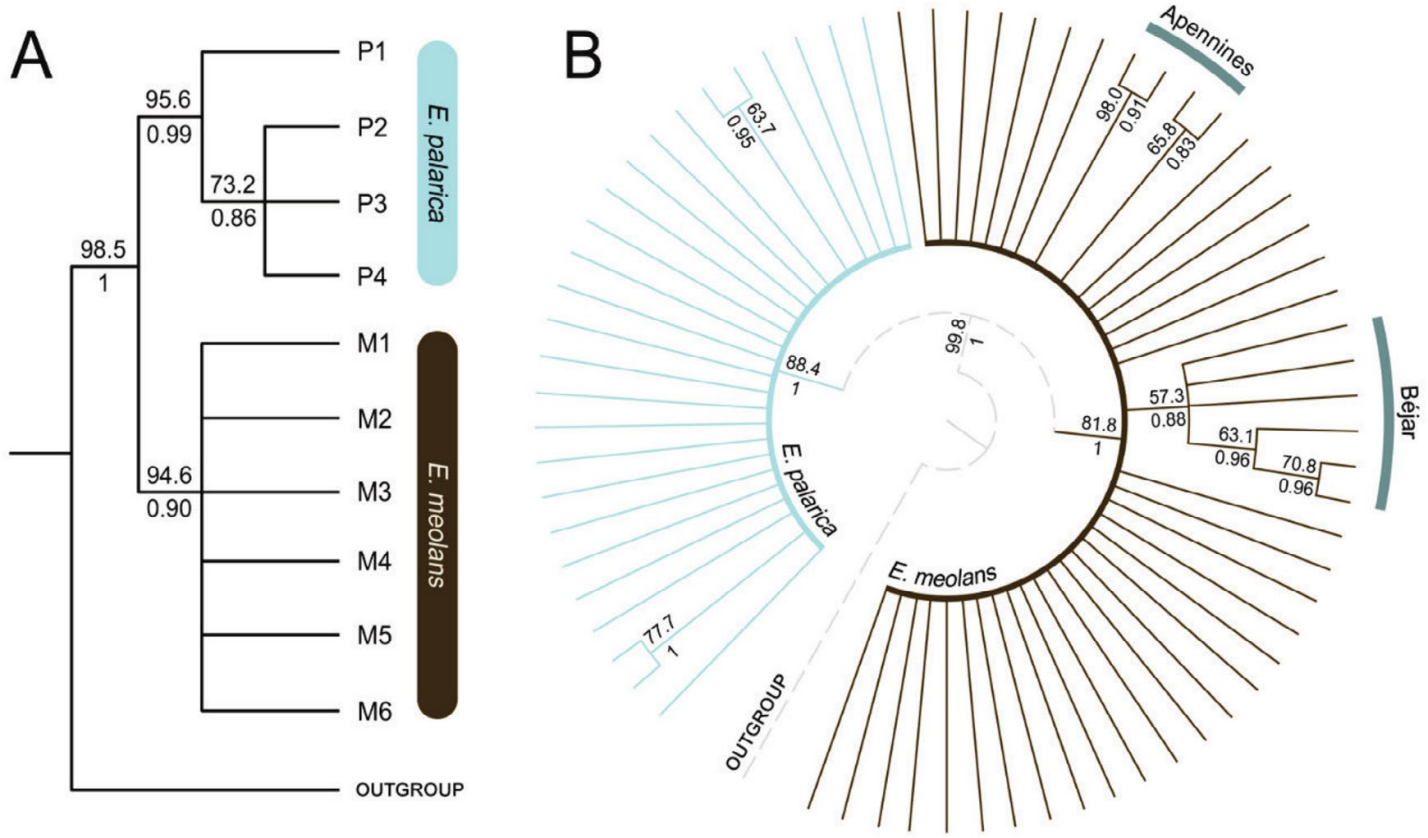
Phylogenetic tree reconstructions for *Erebia meolans* and *Erebia palarica* rooted with ( *E. fletcheri* [*E. triaria*, *E. euryale*]). Consensus trees obtained via ML and BI for the nuclear gene *wingless* (A), and for the concatenated sequences of *wingless* and *COI* (B). Consensus topologies were obtained by collapsing branches with bootstrap values below 55%. In both panels, support is indicated above and below the branch, as given by ML (bootstrap, %) and BI (posterior probability, [0–1]), respectively.

The haplotype network obtained from the 786 bp fragment of the mitochondrial *COI* gene supports a clear distinction between *E. meolans* and *E. palarica* (Figure 4, Figure S3). The two species are separated by eight mutational steps (1.02% sequence divergence) that connect via three tip haplotypes, all present at Somiedo (Asturias, western Cantabrian Mountains; see Torrado‐Blanco et al. (2024) for further details about the haplotype network of *E. palarica*).

**FIGURE 4:**
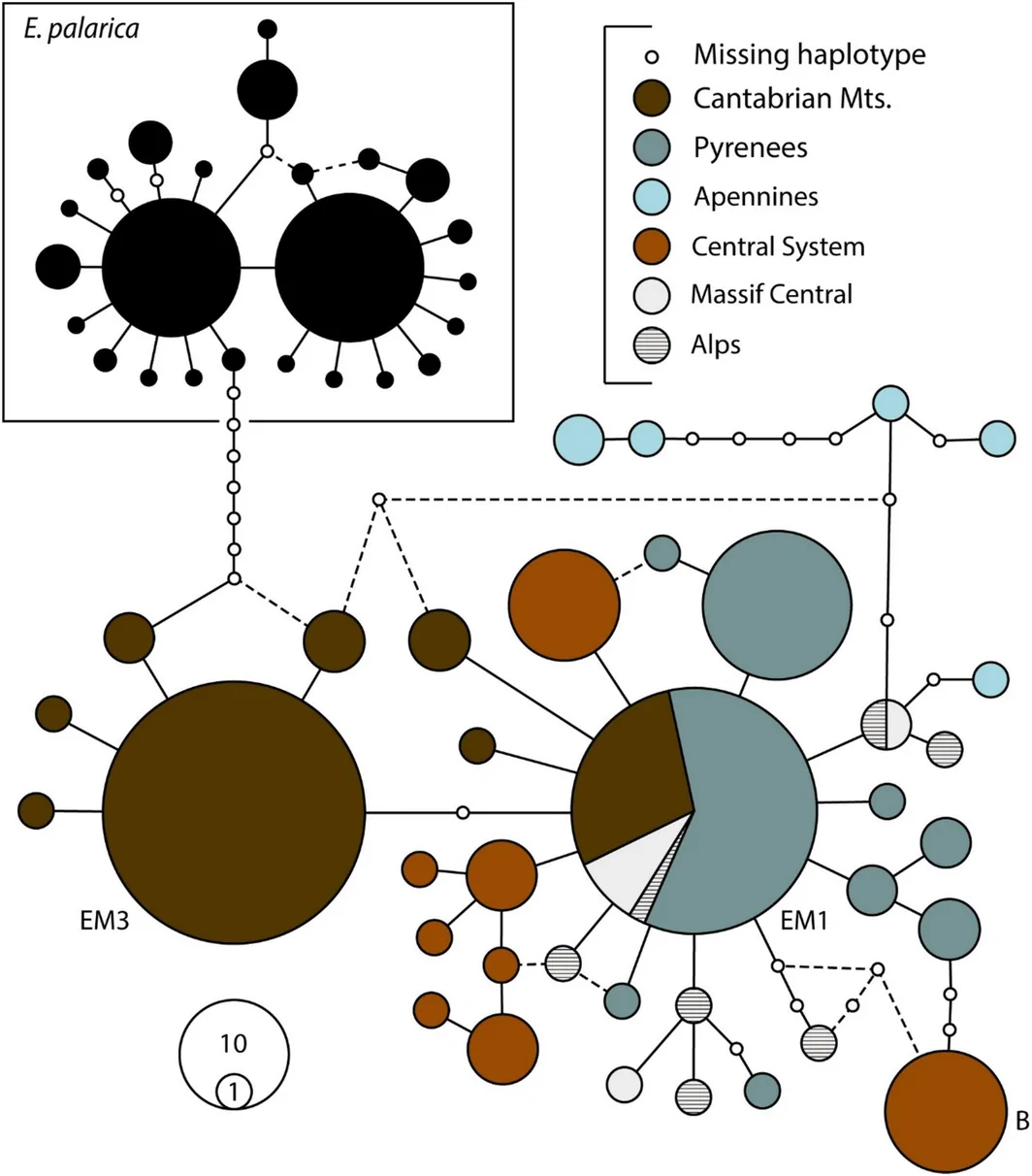
Haplotype network (*COI*, 786 bp). The 95% statistical parsimony haplotype network for the 188 individuals of *Erebia meolans*, connected to 305 *Erebia palarica* samples (black) analysed in a previous study (Torrado‐Blanco et al. 2024). Missing haplotypes are represented as 〇, single mutations as solid lines. Dotted lines indicate the least probable connections in the unresolved loops following the empirical predictions reviewed in Posada and Crandall (2001). Colours correspond to the major mountain ranges across the distribution range of the species (Figure 2). Core haplotypes (EM3, EM1) and samples from Sierra de Béjar (B) are highlighted. The scale at the bottom left indicates the size of pie charts containing 1 and 10 samples, and applies only to *E. meolans* data.

Our dataset revealed 36 *E. meolans* haplotypes, 34 of which are new, and produced four looped connections that were resolved following Posada and Crandall (2001). The network of *E. meolans* shows a double star‐like phylogeographic pattern, with a pair of broadly distributed core haplotypes (EM1, EM3) separated by two mutational steps and connected to several—more geographically restricted—tip haplotypes (EM2, EM4–EM36). Core haplotype EM3 and its satellites are present exclusively across the Cantabrian range. Conversely, haplotype EM1 and derived ones are broadly distributed across Iberia and Central Europe (Figure 4). Two distinct haplogroups arise within this EM1 star: (i) individuals from Sierra de Béjar (central Iberia) are grouped within a single haplotype and separated from the core by five mutational steps; in addition, (ii) five local haplotypes from the Apennines show a separation from the centre of the star of 3–10 mutational steps.

#### 3.1.3 Wolbachia

Both markers (*wsp*, *gatB*) produced a similar pattern. In the case of *E. palarica*, the five allopatric individuals (Galicia, NW Spain) were uninfected by *Wolbachia*, whereas 14 out of the 15 specimens from three Cantabrian sites—where *E. meolans* co‐occurs—were infected by *Wolbachia*. One individual from the Cantabrian Mountains (P16), failed to amplify both genes. With regard to *E. meolans*, 19 out of the 20 specimens proved positive for *Wolbachia* infection, regardless of their geographic origin. Only one individual from the Pyrenees (M02, see Supporting Information 1) failed to amplify both genes despite having good quality DNA. All infected individuals of *E. palarica* and *E. meolans* hosted the same strain of *Wolbachia* according to the *Wolbachia* MLST Database (https://pubmlst.org/bigsdb?db=pubmlst_wolbachia_seqdef): allele 694 for the *wsp* gene (100% identity with Accession Numbers OX366328 and KC137219 as revealed by BLASTN) and allele 9 for *gatB* (100% identity and coverage with many accession numbers, e.g., OX366328).

### 3.2 Chromosome Analysis

We found no typical metaphase meiotic divisions in neither the 11 examined specimens of *E. meolans* nor the 25 specimens of *E. palarica*. This result was expected, since typical meiotic divisions are extremely rare in adult *Erebia* males (Lorković 1990). We found cells undergoing atypical asynaptic meiosis suitable for chromosome counting in four samples of *E. meolans* and five samples of *E. palarica*. In all cases, the count revealed a diploid number of 2*n* = 28. In addition, we found mitotic divisions suitable for chromosome counting in a single individual of *E. palarica*, which also revealed 2*n* = 28. Regarding size variation, the observed chromosomes constitute a gradual range where the first pair is somewhat distinguished by dimension (Figure 5).

**FIGURE 5:**
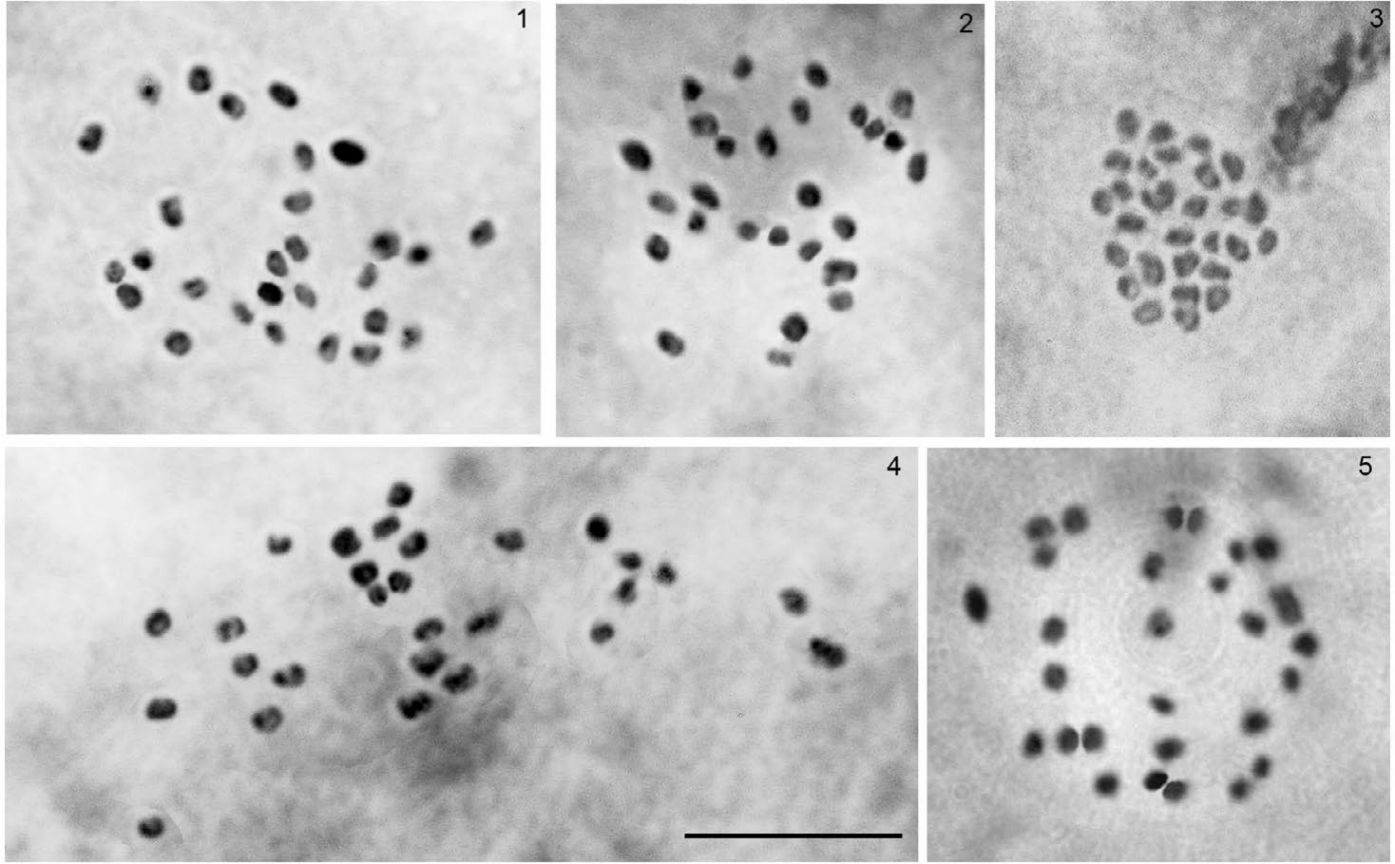
Karyotypes of *Erebia meolans* and *Erebia palarica*. (1) *E. meolans*, sample EM_SET04, male asynaptic meiosis, 2*n* = 28; (2) *E. palarica*, EP_TRE0802, male asynaptic meiosis, 2*n* = 28; (3) *E. palarica*, EP_TRE0607, male, mitosis, 2*n* = 28; (4) *E. palarica*, EP_TRE0808, male asynaptic meiosis, 2*n* = 28; (5) *E. palarica*, EP_TRE0819, male asynaptic meiosis, 2*n* = 28. The black bar represents 10 μm. Sample codes refer to those detailed in Supporting Information 1.

### 3.3 Synteny

Not only do *E. meolans* and *E. palarica* share the same karyotype, but they also lack any large‐scale chromosomal rearrangements such as inversions, translocations, fusions or fissions of chromosomes, and their gene order within chromosomes is well conserved. Their genome size is also similar (502.1 Mb for *E. meolans* without considering the W chromosome and 493.2 Mb for *E. palarica*). The only chromosomal rearrangement identified between the reference genomes of the two species is a 16‐gene‐long translocation within chromosome 7 (Figure 6, Table S3).

**FIGURE 6:**
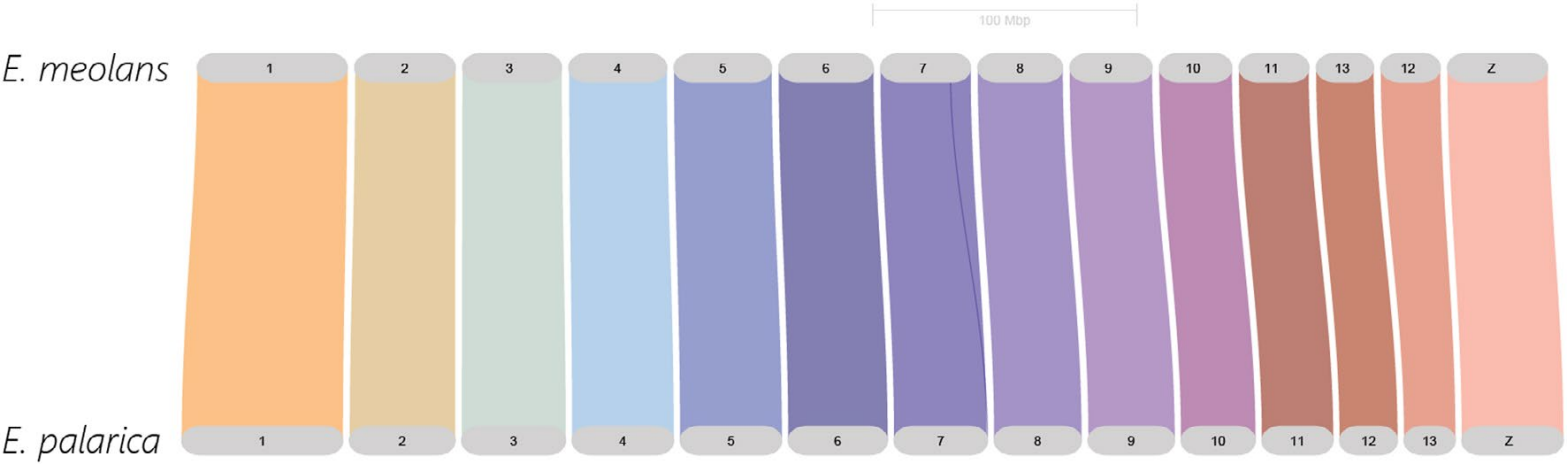
Synteny analysis between the reference genomes of *Erebia meolans* and *Erebia palarica*. Colours match homologous chromosomes in both species. Syntenic blocks are defined as groups of at least five single‐copy orthologous genes kept on the same chromosome and in the same order in both species. Chromosomes are scaled by size. The female‐specific W chromosome, present in the reference genome of *E. meolans*, is not shown.

### 3.4 Morphometric Analysis

#### 3.4.1 Wing Shape

The two leading principal components (PCs) accounted together for 73.6% of the total variance: PC1 (54.3%) is highly correlated with sex differences, whereas PC2 (19.4%) is mostly linked to species (Figure S4).

The MANOVA (Table 2) revealed a significant effect of geographic region (*F*[3,109] = 4.931, *p* < 0.001) and species (*F*[1,111] = 21.720, *p* < 0.001) over wing shape in *E. meolans* and *E. palarica*. The MANCOVA supports this result and, in addition, indicates that the effect of the covariate (i.e., species, *F*[1,108] = 21.352, *p* < 0.001) is stronger than that of geography (*F*[3,108] = 1.744, *p* = 0.024). When analysing the data separately for each species, the MANOVAs reveal a significant effect of sex on wing shape for both *E. meolans* (*F*[1,57] = 5.004, *p* < 0.001) and *E. palarica* (*F*[1,52] = 3.932, *p* = 0.003). No intraspecific shape differences were found when comparing allopatric and sympatric sites on either species: *E. meolans*, *F*[1,57] = 1.247, *p* = 0.292; *E. palarica*, *F*[1,52] = 0.663, *p* = 0.679.

**TABLE 2:** Morphometrics.

Variables	Procedure	Factors			
		Sex		Species	Region
		*Erebia meolans*	*Erebia palarica*		
Shape	GPA‐PCA	**5.004** (< 0.001)	**3.932** (0.003)	**21.720** (< 0.001)	**4.931** (< 0.001)
Size	Centroid size	1.945 (0.057)	−1.264 (0.216)	**−19.681** (< 0.001)	**15.194** (< 0.001)

*Note:* Statistical significance of the morphometric analyses. Differences in group means regarding shape and size were assessed via MANOVAs and *t*‐tests/ANOVAs, respectively (see Section 2 for further details). Associated *p‐*values are indicated in parentheses and significant tests are highlighted in bold. Note that the data of *E. meolans* and *E. palarica* were analysed independently when investigating sex differences, to avoid misleading results due to interspecific variation.

Although these analyses indicate an absence of statistically significant intraspecific shape divergence, the canonical variate analysis (CVA) revealed structured multivariate variation associated with species identity and geographic origin. The most informative linear combinations explained 81.6% of the total variance in wing shape along the two leading axes. The first canonical variate (CV1, 59.6%) clearly separated *E. meolans* and *E. palarica*. Complementarily, CV2 (21.9%) further divides each species into two well‐defined clusters that closely correspond to geographic regions, separating *E. palarica* populations from the Cantabrian Mountains (sympatric) and Galicia (allopatric), and *E. meolans* populations from the Cantabrian Mountains (sympatric) and the Pyrenees (where *E. palarica* is absent) (Figure 7). This division is further supported by CV3 (18.4%).

**FIGURE 7:**
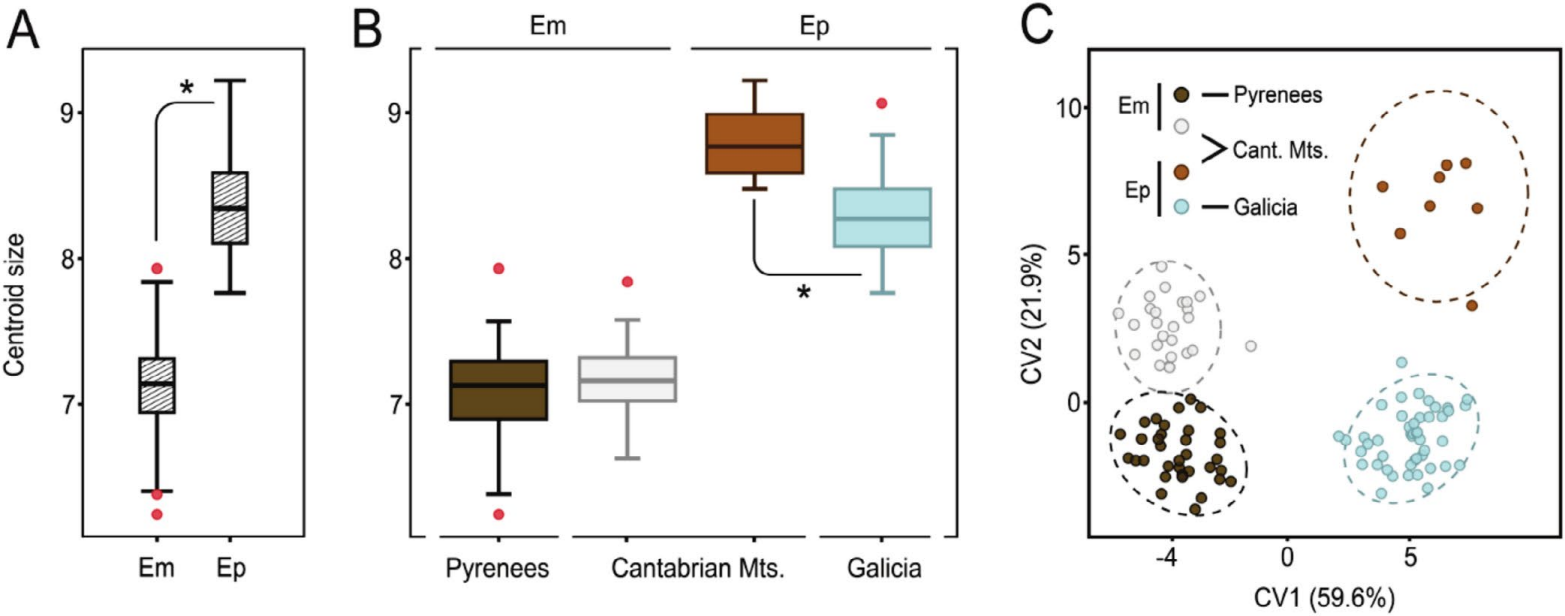
Wing morphometric analyses of sympatric and allopatric *E. meolans* (Em) and *E. palarica* (Ep) from the Iberian Peninsula. Panels show differences in centroid size between species (A) and geographic region (B), and results of the canonical variate analysis of wing shape variation per geographic region (C). Asterisks (*) highlight statistically significant comparisons.

#### 3.4.2 Wing Centroid Size

The *t*‐test of centroid size data (Table 2) revealed a significant effect of species on wing size (*t*[108] = −19.681, *p* < 0.001), with *E. meolans* individuals being remarkably smaller than those of *E. palarica*. With regard to sex, intraspecific *t*‐tests detected no differences between males and females in neither *E. meolans* (*t*[45] = 1.945, *p* = 0.057) nor *E. palarica* (*t*[30] = −1.264, *p* = 0.216).

The one‐way ANOVA suggested significant differences in mean wing centroid size between geographic groups (*F*[3,109] = 15.194, *p* < 0.001). Tukey's HSD further reveals that (Table 3, Figure 7B) (i) the wing centroid size of *E. meolans* and *E. palarica* differs significantly regardless of geographic locality; (ii) allopatric and sympatric populations of *E. meolans* show no evidence of wing size differences; and (iii) allopatric and sympatric populations of *E. palarica* clearly diverge in overall wing size. In other words, there is a clear difference in terms of wing size between Iberian *E. meolans*, being smaller, and *E. palarica* being bigger. The overall size of *E. meolans* is relatively constant across its distribution range in the northern margin of the Iberian Peninsula (*p =* 0.602, 95% CI = −0.316, 0.112). Conversely, allopatric populations of *E. palarica* (i.e., Galicia) are remarkably smaller than those coexisting with *E. meolans* along the Cantabrian Mountains (*p* < 0.001, 95% CI = −0.815, −0.192). Importantly, size differences between both species become more evident in sympatric populations (*p* = 0.001, 95% CI = 1.286, 1.946).

**TABLE 3:** Tukey tests ANOVA.

(a)		Ep	
		Cant. Mts.	Galicia
Em	Cant. Mts.	1.616 (1.285, 1.946); ***p* < 0.001**	1.112 (0.910, 1.314); ***p* < 0.001**
	Pyrenees	−1.717 (−2.037, −1.398); ***p* < 0.001**	−1.213 (−1.397, −1.030); ***p* < 0.001**

(b)		
Em	Cant. Mts./Pyrenees	−0.102 (−0.316, 0.112); *p* = 0.603
Ep	Cant. Mts./Galicia	−0.504 (−0.815, −0.193); ***p* < 0.001**

*Note:* Tukey HSD test of the one‐way ANOVA performed to assess geographic differences in mean centroid size between Iberian *Erebia meolans* and *Erebia palarica* (a) and within each of the aforementioned species (b). Reported values represent the difference in test scores and its associated 95% confidence interval (parentheses) and *p*‐value adjusted for multiple comparisons. Significant *p‐*values are highlighted in bold.

#### 3.4.3 Genitalia

We found no distinct patterns when comparing the size of male and female genitalia of *E. meolans* and *E. palarica* (Supporting Information 1). Anatomical differentiation between the species in terms of the genital apparatus is very subtle, and only noticeable in male structures. Based on our observations, we generated the first detailed illustration representing the typical anatomy of female genitalia in *E. meolans* and *E. palarica* (Figure 8), based on multiple specimens and updating the figure of the genital plates of both species drawn by de Lesse (1949).

**FIGURE 8:**
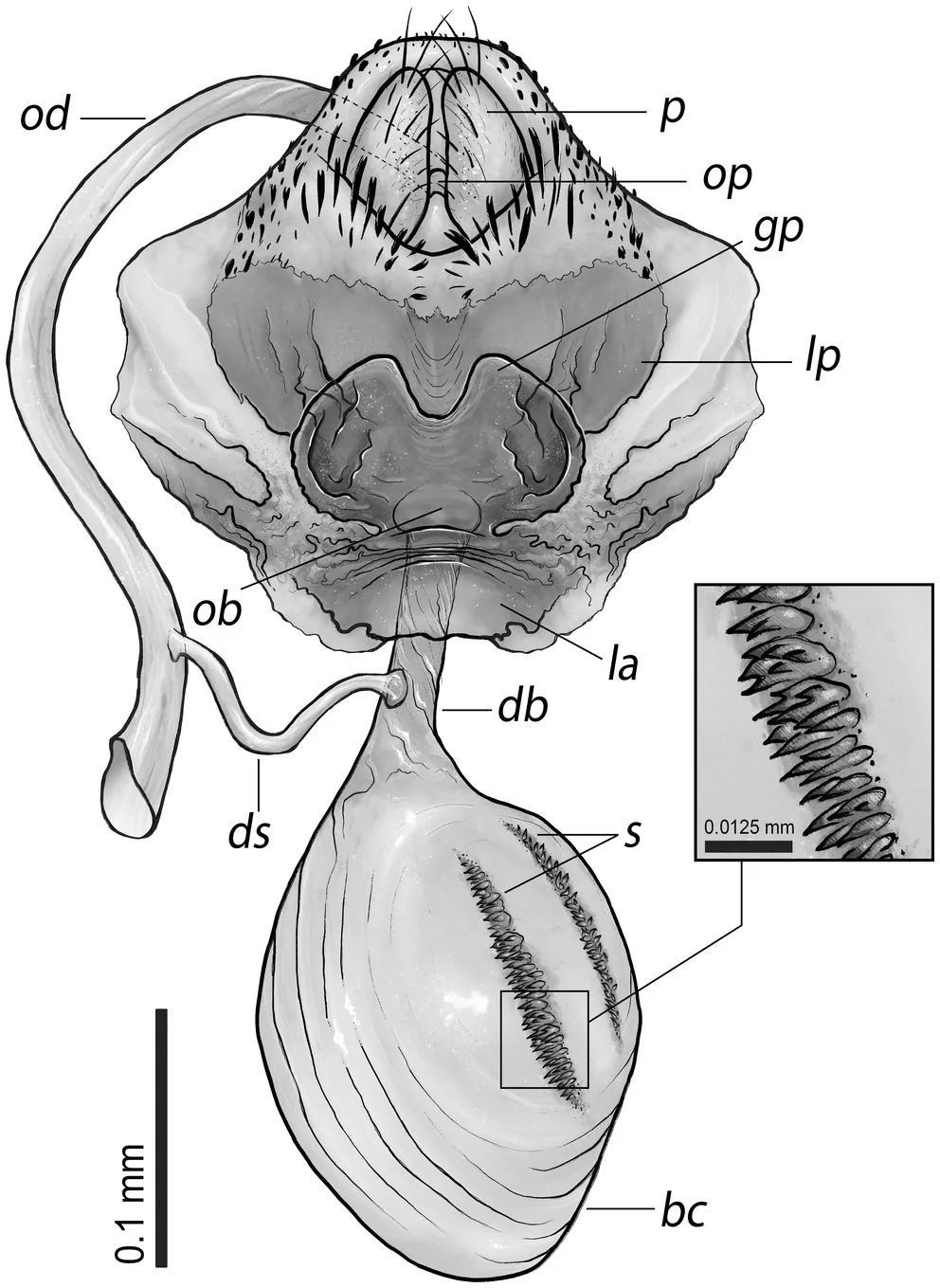
Consensus anatomy of the female genitalia in *Erebia meolans* and *Erebia palarica*. Abbreviations follow García‐Barros et al. (2013) and refer to the *lamella antevaginalis* (*al*), *bursa copulatrix* (*bc*), *ductus bursae* (*db*), *ductus seminalis* (*ds*), genital plate (*gp*), *ostium bursae* (*ob*), oviduct (*od*), ovipore (*op*), genital papillae (*p*), *lamella postvaginalis* (*pl*) and *signa* (*s*). The inset shows a close‐up detail of the denticles.

## 4 Discussion

We investigated the intriguing differentiation case of *E. meolans* and *E. palarica*. These sister taxa are sympatric in the Cantabrian Mountains. However, a question arises with respect to their degree of genetic isolation and, consequently, to their putative reproductive barriers.

### 4.1 No Sign of Recent Introgression

Several lines point to a lack of hybridisation between *E. meolans* and *E. palarica*. The first is the absence of reports of hybrids or interspecific matings registered in the literature despite their similarity and recent divergence (see below). Conversely, there is well‐documented evidence of hybridisation occurring between other closely related *Erebia* taxa. Schmitt and Müller (2007) report interbreeding between two distinct lineages of *E. medusa* ([Denis and Schiffermüller], 1775) in the Hercynian Mountains (central Europe) or between subspecies of *E. euryale* in the Alps (Bouaouina et al. 2023). Hybrid individuals were also detected in a Swiss contact zone between *E. cassioides* and the narrowly distributed *E. nivalis* and *E. tyndarus* (Augustijnen and Lucek 2024; Lucek et al. 2020). Another striking example is the hybrid between *E. epiphron* and *E. pronoe* that used to be described as a distinct taxon *E. serotina* Descimon & de Lesse, 1953, discussed in Michel et al. (2013). Lastly, a hybrid produced by a male *E. meolans* and a female *E. pronoe* (two species that are much less closely related) has been found in the Pyrenees (Lalanne‐Cassou et al. 2018).

Second, the reciprocal monophyly recovered by the nuclear and mitochondrial markers suggests the absence of recent hybridisation between *E. meolans* and *E. palarica* (Figure 3). Introgression and hybridisation leave traces in the genetic makeup of species and can thus be detected even with a few markers if the events are relatively recent (Page and Holmes 1998). This has been demonstrated in other species using the same (Kosuda et al. 2016), or similar (Zakharov et al. 2009) molecular markers used in this study.

Third, the single connection—eight mutational steps apart—between *E. meolans* and *E. palarica* in the haplotype network further supports the hypothesis of absence of interbreeding. Such a genetic link occurs between haplotypes currently distributed in Somiedo (western part of the Cantabrian Mountains), an area where both species currently overlap. Indeed, the pattern reflected by our haplotype network is very different to that found in cases where introgression occurs. In such scenarios, haplotype networks usually reveal multiple looped connections or shared haplotypes between taxa (e.g., Boratyński et al. 2011; Corrigan et al. 2017; Zhigileva et al. 2020; Nge et al. 2021; Li et al. 2022).

The phylogenetic congruence between the mitochondrial and nuclear markers, the high degree of differentiation revealed by *F*_ST_, and the haplotype network indicate a strong reproductive isolation between Piedmont and Chapman's ringlets. Nevertheless, we are aware that this putative lack of introgression has to be tested by future genomics studies.

### 4.2 No Evidence for a Role of *Wolbachia* in Reproductive Isolation

An increasing number of studies have highlighted the role of *Wolbachia* infections in promoting reproductive isolation among insect species (Ahmed et al. 2015; Bella et al. 2010; Bordenstein et al. 2001; Miller et al. 2010; Nice et al. 2009; Sasaki and Ishikawa 1999), and, specifically, *Erebia* butterflies (Augustijnen and Lucek 2024; Lucek et al. 2021). However, our results suggest that *Wolbachia* is unlikely to contribute to reproductive isolation between the sympatric populations of *E. meolans* and *E. palarica* studied here.

We detected a high prevalence of *Wolbachia* in sympatric populations of *E. palarica* and *E. meolans*, whereas *E. palarica* individuals from allopatric sites (i.e., Galicia) were clearly non‐infected by this endosymbiont. Notably, all infected individuals shared the same *Wolbachia* strain, regardless of the species and geographic location. This pattern suggests that reproductive isolation in the sympatric populations of both species is not driven by the presence of *Wolbachia*, as no cytoplasmic incompatibilities would occur in the event of interbreeding. *Wolbachia* infection in *E. palarica* may have been acquired in the contact zone with *E. meolans*, which is infected throughout all surveyed sites in the Iberian Peninsula. The most parsimonious explanation for this scenario would be a recent transfer of *Wolbachia* from *E. meolans* to *E. palarica* in sympatry. The situation resembles that of the pair *Iphiclides podalirius* and *I. feisthamelii*, where transspecific infection of *Wolbachia* has also been proposed (Gaunet et al. 2019). Transspecific *Wolbachia* infection does not necessarily imply vertical transmission via hybridisation, as horizontal transfer might result from parasitoid or ectoparasite attack, as well as feeding [reviewed by Shastry et al. (2022)]. Further studies should test whether any cytoplasmic incompatibility exists between infected Cantabrian populations of *E. palarica* (sympatric with *E. meolans*) and the uninfected allopatric populations from Galicia.

### 4.3 Conserved Karyotype

Changes in chromosome number resulting from fusion and fission events represent a key mechanism driving butterfly speciation, particularly in the genus *Erebia* (Augustijnen et al. 2024). However, we found no evidence that chromosomal rearrangements contribute to the differentiation between *E. palarica* and *E. meolans*.

We determined the karyotype of *E. palarica* to be 2*n* = 28 based on cytogenetic analyses, in agreement with the chromosome‐resolved reference genome assembly (Vila et al. 2026). Such number equals the one described for *E. meolans* by previous chromosome counts (de Lesse 1953a, 1953b, 1960, 1961; Robinson 1971), being also congruent with our own observations. *Erebia* butterflies exhibit a remarkable interspecific karyotypic variability, with chromosome numbers ranging from 2*n* = 14 to 2*n* = 104 (Robinson 1971). The number 2*n* = 58 (*n* = 29) is modal for the genus and can be interpreted as the ancestral karyotype. Thus, it appears that *E. meolans* and *E. palarica* share the derived chromosome number 2*n* = 28, which can be interpreted as a cytological synapomorphy of the species pair. Augustijnen et al. (2024) modelled chromosome number evolution in the genus *Erebia* and not only estimated a karyotype of 2*n* = 28 for *E. palarica*, but also provided similar chromosome count estimations for other *Erebia* within the *medusa* species group. The karyotype 2*n* = 28 is also found in *E. oeme* (Hübner, 1803–1804) (Lorković 1941; Robinson 1971). Interestingly, this butterfly belongs to the distantly related *pronoe* species group (Augustijnen et al. 2024; Peña et al. 2015; Wiemers et al. 2020), which suggests that the karyotype 2*n* = 28 has likely evolved at least twice independently in the genus.

The differentiation of butterfly species with the same number of chromosomes may be driven by intrachromosomal rearrangements, such as inversions, translocations, or multiple chromosomal fusions and fission events (Höök et al. 2023; Pazhenkova and Lukhtanov 2023). Our synteny analysis suggests that such rearrangements are nearly absent (Figure 6) and therefore likely do not constitute a strong barrier to gene flow between *E. palarica* and *E. meolans*. However, it should be noted that the chromosome‐level reference genomes for these sister species were generated from allopatric populations (Galicia and Alps, respectively). Therefore, we cannot rule out the presence of genomic rearrangements in sympatric individuals, as karyotypic differences are known to accumulate more rapidly between sympatric than allopatric sister taxa in other chromosomally diverse phylogenetic lineages (Kandul et al. 2007).

### 4.4 Phylogeography, Genetic Diversity and the Origin of Sympatry

Species with narrow distribution ranges usually harbour lower genetic diversity than those broadly distributed (Frankham 1996; Frankham et al. 2010). This tendency is reflected by our studied species, as we found slightly higher diversity levels in *E. meolans* than in *E. palarica* (Table 1). Interestingly, the genetic makeup of both species appears to be considerably diverse when compared to other European Lepidoptera—for example *E. triaria* (Vila et al. 2005); *E. euryale* (Vila et al. 2011); *Hyles euphorbiae* (Linnaeus, 1758) (Mende et al. 2016).

With regard to the genetic structure of *E. meolans*, samples from Sierra de Béjar (Central Spain) and central Apennines stand out as the most differentiated in both the phylogenetic trees and the haplotype network. These sites are located at the southwestern (Béjar) and southeastern (Apennines) limits of the distribution range of *E. meolans* and correspond to small, geographically isolated mountain systems. Such peripheral populations often exhibit reduced genetic diversity and increased differentiation (Eckert et al. 2008). Indeed, several studies have reported the presence of highly divergent—and often threatened—butterfly lineages in the central Apennines (Bereczki et al. 2022; Bonifacino et al. 2022; Menchetti et al. 2021; Sistri et al. 2022). Moreover, *E. meolans* from Sierra de Béjar were described as subspecies *bejarensis*, although the taxonomic validity of this designation remains uncertain (García‐Barros et al. 2013). From a conservation perspective, the pronounced genetic differentiation of these southernmost populations is particularly relevant, as their potential loss under ongoing climate change would result in a disproportionate erosion of species‐level genetic diversity, a pattern previously highlighted in other *Erebia* species (Hinojosa et al. 2019).

The mitochondrial network obtained for each species shows a characteristic double star‐like pattern (Torrado‐Blanco et al. 2024; Figure 4), a configuration likely derived from population expansions during the last glacial cycle, as reported in other European *Erebia* (Albre et al. 2008; Cupedo and Doorenweerd 2022; Vila et al. 2005). Presumably, the two core lineages within each species (*E. palarica*: EP0, EP1; *E. meolans*: EM1, EM3) underwent postglacial population and range expansions, coming into secondary contact as the four of them colonised the Cantabrian Mountains. This double colonisation of the Cantabrian Mountains from unknown refugial areas led to the current sympatry of *E. palarica* and *E. meolans*.

The mitochondrial haplotype network also suggests that *E. palarica* derives from a common ancestor meolans/palarica whose descendants currently inhabit Somiedo (western Cantabrian Mountains). Augustijnen et al. (2024) estimate the approximate time of this divergence at ≈0.773 My BP, while Klečková et al. (2023) suggest an earlier split, around ≈2.9 My BP. Altogether, it appears that the divergence of the species pair occurred sometime during the early Pleistocene and was likely heavily influenced by the climatic instability typical of that period. In addition, we hypothesise that the EM1 lineage of *E. meolans*—core haplotype and derived ones, present across the whole distribution range—is the ancestral one. This is because the genus *Erebia* originated in Asia and colonised Europe dispersing westwards (Peña et al. 2011, 2015). Thus, core haplotype EM3 and derived ones –i.e. the lineage of *E. meolans* more closely related to *E. palarica* and exclusive to the Cantabrian Mountains—likely diverged from this ancestral line (Figure 4 and Figure S3).

### 4.5 Morphology and Character Displacement

The reciprocal monophyly obtained from both mitochondrial and nuclear genetic markers suggests the presence of a clear barrier to genetic exchange between *E. palarica* and *E. meolans*. Female genital morphology, *Wolbachia* infection and chromosomal rearrangements do not show a clear signal of divergence between the two species, which is consistent with these factors playing a limited role as reproductive barriers to interspecific gene flow, although the modest sample sizes available for genital comparisons mean this absence of detected differences cannot be taken as conclusive. In the light of the geometric morphometric results, we hypothesise that selection associated with reinforcement may have contributed to wing size divergence, resulting in character displacement that could, in turn, be acting as a reproductive barrier between the two species.

Several criteria must be met in order to support character displacement: (i) the studied pair of species must be phylogenetically close, phenotypically similar and have clear sympatric and allopatric distributions; (ii) the effects of coexistence should be clearly distinguished from other external or ecological factors; (iii) the state of the displaced trait must differ between sympatric and allopatric populations of the interacting taxa; and (iv) such difference must be most pronounced where both species overlap (Futuyma 1998; Kawano 2003).

The enlarged wing size of *E. palarica* relative to sympatric *E. meolans* populations is consistent with a scenario of asymmetric character displacement: *E. meolans* shows no intraspecific size variation in our Cantabrian sample, while *E. palarica* from the Cantabrian Mountains (i.e., sympatric) have significantly larger wings than conspecifics from western allopatric sites, resulting in the strongest interspecific size differences in the area where both butterflies coexist (Table 3). The star‐like patterns revealed by the haplotype networks suggest that the different lineages of *E. meolans* and *E. palarica* diverged in allopatry, and recolonised the Cantabrian Mountains after the Last Glacial Maximum. Thus, the observed size displacement in *E. palarica* is likely a response to this secondary contact, driven by selection, to avoid maladaptive hybridisation.

The asymmetry of the observed character displacement could be explained by several factors, reviewed in Cooley (2007). First, differences in hybrid cost between interbreeding species may impose stronger selection on one of them, leading to asymmetries in the affected character. Second, biological constraints in the displaced trait—here, wing size—may limit the range of phenotypic variation. Both species are known to vary in wing size: (a) *E. palarica* has larger wings along the Cantabrian Mountains; (b) *E. meolans bejarensis* has larger wings than other Spanish populations (García‐Barros et al. 2013; Manley and Allcard 1970); (c) Higgins (1975) mentions a cline in the overall size of *E. meolans* subspecies, which become larger in size in the Iberian Peninsula, as compared to the smaller individuals found in Central Europe; and (d) our results here show that some *E. meolans* from Coll de Jou (Pyrenees) have larger wings than the rest. Together, these observations suggest that Cantabrian *E. meolans* may already lie close to the biological lowest wing size limit possible for this species, potentially constraining further size reduction. Finally, a scenario involving the extinction of larger *E. meolans* populations seems unlikely, given that the ancestral haplotype is present across the Pyrenees, that is the allopatric areas. Asymmetric and divergent wing size character displacement has also been previously reported in *Pyrgus* butterflies (de Jong 1979) and suggested in *Saturnia* moths (Khan et al. 2024). Character displacement affecting other wing traits has been previously reported in butterflies (i.e., colour shifts in *Heliconius* spp. [reviewed by Joron et al. 2006] and *Cupido* spp. [Hinojosa et al. 2020]). However, and to the best of our knowledge, ours is the first study using morphometrics to determine wing size character displacement in a butterfly, and the first one to report this phenomenon within the family Nymphalidae.

Assuming a positive correlation between wing size and body size (Miller 1977), we initially hypothesised that reinforcement of size differences in sympatry could limit admixture via mechanical incompatibilities of the genitalia. This hypothesis was based on prior reports of genital incompatibility or tactile rejection preventing interspecific matings in *Erebia* (Lorković 1958; Coyne and Orr 2004, 229). However, our observations point towards substantial intraspecific size variation for both male and female genitalia in both species, which is more consistent with an absence of strong mechanical incompatibility than with its presence. We note, however, that our genital sample sizes were small, and we did not perform a formal power analysis; as such, we cannot rule out subtle differences that a larger sample might reveal, and this hypothesis should be revisited with expanded sampling.

Species interactions are complex and multifactorial, and other barriers separating *E. meolans* and *E. palarica* are likely in place. Notably, the increase in wing size observed in sympatric *E. palarica* is unlikely to result from plastic responses to interspecific competition or habitat exclusion, as such processes typically lead to reduced body size through developmental or dietary constraints. Nevertheless, the observed displacement in overall size does not necessarily imply that this trait is itself under direct selection. Other key factors contributing to reproductive isolation (e.g., chemical signalling or courtship behaviour) may be under selection, with genes involved in wing size responding indirectly via genetic hitchhiking; for example genes for pterin coloration and wing size are Z‐linked in *Colias* butterflies (Hanly et al. 2023). In addition, habitat specialisation is one of the mechanisms facilitating coexistence of *Erebia* species (Klečková et al. 2014). Knowledge of the Poaceae host plants used by *E. meolans* and *E. palarica* in the Cantabrian Mountains remains limited (Clarke 2024). In this region, *E. meolans* is frequently observed near limestone outcrops (H. Mortera, pers. comm.), whereas *E. palarica* typically occupies grasslands and shrub‐dominated areas associated with acid substrates (Mortera 2008). However, these two microhabitats occur in close proximity, often separated by only a few metres, suggesting that interspecific encounters and courtship remain plausible despite a microhabitat association.

The case of *E. meolans* and *E. palarica* underscores the value of integrative morphological and genomic approaches for understanding speciation, species limits and intraspecific diversity. Together, these results provide a framework for future genomic studies aimed at testing for signatures of introgression and identifying the genetic basis of species differentiation in the absence of genomic rearrangements.

## Author Contributions

**Laura Torrado‐Blanco:** funding acquisition (supporting), investigation (lead), visualization (lead), writing – original draft (lead). **Roger Vila:** conceptualization (supporting), investigation (supporting), visualization (supporting), writing – original draft (supporting). **Vladimir A. Lukhtanov:** funding acquisition (supporting), investigation (equal), visualization (supporting), writing – original draft (equal). **Carmen Seoane‐Rodríguez:** investigation (supporting), visualization (supporting), writing – review and editing (supporting). **Camille Cornet:** investigation (equal), visualization (supporting), writing – original draft (equal). **Kay Lucek:** funding acquisition (supporting), investigation (supporting), writing – original draft (supporting). **Marta Vila:** conceptualization (lead), funding acquisition (lead), investigation (lead), project administration (lead), writing – original draft (equal).

## Funding

This work was supported by grants from Xunta de Galicia (PGIDIT06PXIB103258PR, ED431C 2018/57) and Universidade da Coruña. Laura Torrado‐Blanco was funded by Xunta de Galicia (ED481A/2020‐300). Chromosomal studies were funded by the Russian Science Foundation Grant No. 24‐14‐00047 (https://rscf.ru/project/24‐14‐00047) to Vladimir A. Lukhtanov. Camille Cornet and Kay Lucek were supported by the Swiss National Science Foundation (PCEFP3_202869).

## Conflicts of Interest

The authors declare no conflicts of interest.

## Supporting information


**Figure S1:** Phylogenetic tree ML COI + wingless: ML phylogenetic tree reconstruction of the concatenated sequences of *wingless* and COI in *E. meolans* and *E. palarica*. Branch support is indicated by bootstrap values (%) next to each node. Bold segments highlight the supported clades mentioned in the principal text: *E. palarica*, *E. meolans*, Sierra de Béjar, Central Apennines, Picos de Europa (E Cant.) and Serra de Ancares (NW Ib.). Outgroup species correspond to *E. euryale* (ITA09), *E. triaria* (OUT01) and *E. fletcheri* (OUT05). Note that sequences MEOPIE1t and MEOPIE1c represent two possible alleles present in a heterozygote butterfly from Piedras Luengas. The aforementioned individual was originally mislabelled as *E. meolans* (hence the “MEO” in the code). However, we double‐checked the identification as *E. palarica* using DNA, genitalia and wing morphology/pattern.
**Figure S2:** Phylogenetic trees BI COI + wingless: BI phylogenetic tree reconstruction of the concatenated sequences of *wingless* and COI in *E. meolans* and *E. palarica*. Branch support is indicated by posterior probability values (0–1) next to each node. Bold segments highlight the supported clades mentioned in the principal text: *E. palarica*, *E. meolans*, Sierra de Béjar, Central Apennines, Picos de Europa (E Cant.) and Serra de Ancares (NW Ib.). Outgroup species correspond to *E. euryale* (ITA09), *E. triaria* (OUT01) and *E. fletcheri* (OUT05). The reader is referred to notes in Figure S1 for further details on sample “MEOPIE1”.
**Figure S3:** Haplotype network: Further information regarding the 95% statistical parsimony haplotype network of 188 *E. meolans*, with *E. palarica* (asterisk, *) as external reference. Codes and numbers correspond to the 36 identified *E. meolans* haplotypes and two additional samples from the Pyrenean locality of Coll de Jou (EM_JOU1, EM_JOU2, relevant for the Discussion). The localities where each haplotype is present can be consulted in tab “Haplotype sites” at Supplementary_spreadsheet_S03_version2.xlsx. Missing haplotypes are represented as “〇,” single mutations as solid lines. Dotted lines indicate the least probable connections in the loops following the empirical predictions reviewed in Posada and Crandall (2001). The scale at the bottom left indicates the size of circles containing 1 and 10 samples as reference.
**Figure S4:** Shape PCA: Principal component analyses of the morphometric data (wing shape) as depicted by sex (A) and species (B).

**Data S1:** ece374533‐sup‐0002‐DataS1.xlsx.

**Table S1:** Sequence availability. Accession numbers of the mitochondrial (*COI*) and nuclear (*wingless*) haplotypes of *Erebia palarica* (Ep) and *E. meolans* (Em). Haplotypes that were newly identified in the present study are highlighted in bold. The reader is referred to Torrado‐Blanco et al. (2024) for further details regarding *COI* haplotypes of *E. palarica* (i.e., EP0‐EP23). All sequences are deposited and publicly available in GenBank/ENA.
**Table S2:** Material used for chromosomal analysis. Details for the 36 adult males of *E. palarica* and *E. meolans* that were dissected for chromosomal preparations. All samples were collected within the Iberian Peninsula (Spain). The abbreviation “ca” (circa) means that the count was made with approximation due to overlapping of some chromosomes.

**Table S3:** Syntenic block and region coordinates.

## Data Availability

All samples were collected receiving prior and explicit approval from the competent authorities. Sequences are publicly available and can be accessed through GenBank (see Table S1).
