# Supplementary material for: Siblings in Flight: Unravelling Reproductive Barriers Between Two Butterfly Sister Species
Source: Ecol Evol. 2026 Nov 8;16(11):e74533. doi: 10.1002/ece3.74533 (PMC13744881; doi:10.1002/ece3.74533)
Supplement: Supplementary file 1 [file ECE3-16-e74533-s001.docx]

**Table S1 – Sequence availability.** Accession numbers of the mitochondrial (*COI*) and nuclear (*wingless*) haplotypes of *Erebia* *palarica* (Ep) and *E. meolans* (Em). Haplotypes that were newly identified in the present study are highlighted in bold. The reader is referred to Torrado-Blanco *et al*. (2024) for further details regarding *COI* haplotypes of *E. palarica* (i.e. EP0-EP23). All sequences are deposited and publicly available in GenBank/ENA.

|  | **Species** | **Haplotype** | **Accession number** |
| --- | --- | --- | --- |
| *wingless* | Ep | **XXX** | XXXXX |
|  | Ep | **XXX** | XXXXX |
|  | Ep | **XXX** | XXXXX |
|  | Ep | **XXX** | XXXXX |
|  | Em | **XXX** | XXXXX |
|  | Em | **XXX** | XXXXX |
|  | Em | **XXX** | XXXXX |
|  | Em | **XXX** | XXXXX |
|  | Em | **XXX** | XXXXX |
|  | | | |
| *COI* | Em | EM1 | AY350455.1 |
|  | Em | EM2 | AY346223.1 |
|  | Em | **EM3** | XXXXX |
|  | Em | **EM4** | XXXXX |
|  | Em | **EM5** | XXXXX |
|  | Em | **EM6** | XXXXX |
|  | Em | **EM7** | XXXXX |
|  | Em | **EM8** | XXXXX |
|  | Em | **EM9** | XXXXX |
|  | Em | **EM10** | XXXXX |
|  | Em | **EM11** | XXXXX |
|  | Em | **EM12** | XXXXX |
|  | Em | **EM13** | XXXXX |
|  | Em | **EM14** | XXXXX |
|  | Em | **EM15** | XXXXX |
|  | Em | **EM16** | XXXXX |
|  | Em | **EM17** | XXXXX |
|  | Em | **EM18** | XXXXX |
|  | Em | **EM19** | XXXXX |
|  | Em | **EM20** | XXXXX |
|  | Em | **EM21** | XXXXX |
|  | Em | **EM22** | XXXXX |
|  | Em | **EM23** | XXXXX |
|  | Em | **EM24** | XXXXX |
|  | Em | **EM25** | XXXXX |
|  | Em | **EM26** | XXXXX |
|  | Em | **EM27** | XXXXX |
|  | Em | **EM28** | XXXXX |
|  | Em | **EM29** | XXXXX |
|  | Em | **EM30** | XXXXX |
|  | Em | **EM31** | XXXXX |
|  | Em | **EM32** | XXXXX |
|  | Em | **EM33** | XXXXX |
|  | Em | **EM34** | XXXXX |
|  | Em | **EM35** | XXXXX |
|  | Em | **EM36** | XXXXX |

**Table S2 –** **Material used for chromosomal analysis.** Details for the 36 adult males of *E. palarica* and *E. meolans* that were dissected for chromosomal preparations. All samples were collected within the Iberian Peninsula (Spain). The abbreviation “ca” (circa) means that the count was made with approximation due to overlapping of some chromosomes.

|  | **Sample** | **Province** | **Locality** | **Result of chromosome analysis** |
| --- | --- | --- | --- | --- |
| *E. meolans* | EM_VIE01 | Lleida | Val d'Aran | 2n=28 (asynaptic meiosis) |
|  | EM_PIT10 | Lleida | Val d'Aran | no divisions |
|  | EM_PIT11 | Lleida | Val d'Aran | no divisions |
|  | EM_PIT12 | Lleida | Val d'Aran | 2n=ca 28 (asynaptic meiosis) |
|  | EM_PIT13 | Lleida | Val d'Aran | no divisions |
|  | EM_BON02 | Lleida | Port de la Bonaigua | no divisions |
|  | EM_BON03 | Lleida | Port de la Bonaigua | no divisions |
|  | EM_SET04 | Girona | Setcasés | 2n=28 (asynaptic meiosis) |
|  | EM_SET05 | Girona | Setcasés | no divisions |
|  | EM_SET07 | Girona | Setcasés | 2n=ca 28 (asynaptic meiosis) |
|  | EM_PIT08 | Lleida | Val d'Aran | no divisions |
|  | | | | |
| *E. palarica* | EP_ANC0801 | Lugo | Ancares | no divisions |
|  | EP_ANC0802 | Lugo | Ancares | no divisions |
|  | EP_ANC0803 | Lugo | Ancares | no divisions |
|  | EP_ANC0804 | Lugo | Ancares | no divisions |
|  | EP_ANC0805 | Lugo | Ancares | no divisions |
|  | EP_ANC0806 | Lugo | Ancares | no divisions |
|  | EP_ANC0807 | Lugo | Ancares | 2n=28 (asynaptic meiosis) |
|  | EP_ANC0808 | Lugo | Ancares | no divisions |
|  | EP_ANC0809 | Lugo | Ancares | no divisions |
|  | EP_ANC0810 | Lugo | Ancares | no divisions |
|  | EP_ANC0811 | Lugo | Ancares | no divisions |
|  | EP_ANC0813 | Lugo | Ancares | no divisions |
|  | EP_TRE0801 | Ourense | Trevinca | no divisions |
|  | EP_TRE0802 | Ourense | Trevinca | 2n=28 (asynaptic meiosis) |
|  | EP_TRE0603 | Ourense | Trevinca | no divisions |
|  | EP_TRE0605 | Ourense | Trevinca | no divisions |
|  | EP_TRE0607 | Ourense | Trevinca | 2n=28 (mitosis) |
|  | EP_TRE0808 | Ourense | Trevinca | 2n=ca 27-28 (asynaptic meiosis) |
|  | EP_TRE0811 | Ourense | Trevinca | 2n=28 (asynaptic meiosis) |
|  | EP_TRE0814 | Ourense | Trevinca | no divisions |
|  | EP_TRE0816 | Ourense | Trevinca | no divisions |
|  | EP_TRE0817 | Ourense | Trevinca | no divisions |
|  | EP_TRE0818 | Ourense | Trevinca | no divisions |
|  | EP_TRE0819 | Ourense | Trevinca | 2n=28 (asynaptic meiosis) |
|  | EP_TRE0821 | Ourense | Trevinca | no divisions |
