# Supplementary material for: Siblings in Flight: Unravelling Reproductive Barriers Between Two Butterfly Sister Species
Source: Ecol Evol. 2026 Nov 8;16(11):e74533. doi: 10.1002/ece3.74533 (PMC13744881; doi:10.1002/ece3.74533)
Supplement: Supplementary file 2 [file ECE3-16-e74533-s003.docx]

**Figure S1. Phylogenetic tree ML COI+wingless:** ML phylogenetic tree reconstruction of the concatenated sequences of *wingless* and COI in *E. meolans* and *E. palarica*. Branch support is indicated by bootstrap values (%) next to each node. Bold segments highlight the supported clades mentioned in the principal text: *E. palarica, E. meolans,* Sierra de Béjar, Central Apennines, Picos de Europa (E Cant.) and Serra de Ancares (NW Ib.). Outgroup species correspond to *E. euryale* (ITA09), *E. triaria* (OUT01) and *E. fletcheri* (OUT05). Note that sequences MEOPIE1t and MEOPIE1c represent two possible alleles present in a heterozygote butterfly from Piedras Luengas. The aforementioned individual was originally mislabelled as *E. meolans* (hence the “MEO” in the code). However, we double-checked the identification as *E. palarica* using DNA, genitalia and wing morphology/pattern.

**Figure S2. Phylogenetic trees BI COI+wingless:** BI phylogenetic tree reconstruction of the concatenated sequences of *wingless* and COI in *E. meolans* and *E. palarica*. Branch support is indicated by posterior probability values (0‒1) next to each node. Bold segments highlight the supported clades mentioned in the principal text: *E. palarica, E. meolans,* Sierra de Béjar, Central Apennines, Picos de Europa (E Cant.) and Serra de Ancares (NW Ib.). Outgroup species correspond to *E. euryale* (ITA09), *E. triaria* (OUT01) and *E. fletcheri* (OUT05). The reader is referred to notes in Figure S1 for further details on sample “MEOPIE1”.

**Figure S3 ‒ Haplotype network**: Further information regarding the 95% statistical parsimony haplotype network of 188 *E. meolans*, with *E. palarica* (asterisk, *) as external reference. Codes and numbers correspond to the 36 identified *E. meolans* haplotypes and two additional samples from the Pyrenean locality of Coll de Jou (EM_JOU1, EM_JOU2, relevant for the Discussion). The localities where each haplotype is present can be consulted in tab “Haplotype sites” at Supplementary_spreadsheet_S03_version2.xlsx. Missing haplotypes are represented as “〇”, single mutations as solid lines. Dotted lines indicate the least probable connections in the loops following the empirical predictions reviewed in Posada & Crandall (2001). The scale at the bottom left indicates the size of circles containing 1 and 10 samples as reference.

**Figure S4 ‒ Shape PCA**: Principal component analyses of the morphometric data (wing shape) as depicted by sex (A) and species (B).
